# A humanized HLA-DR4 mouse model for autoimmune myocarditis

**DOI:** 10.1016/j.yjmcc.2017.04.003

**Published:** 2017-06

**Authors:** M. Emrah Şelli, Anita C. Thomas, David C. Wraith, Andrew C. Newby

**Affiliations:** a University of Bristol, School of Clinical Sciences, UK; b Bristol Heart Institute, Bristol Royal Infirmary, Bristol BS2 8HW, UK; c University of Bristol, School of Cellular and Molecular Medicine, University Walk, Bristol BS8 1TD, UK

**Keywords:** Autoimmunity, Myocarditis, Cardiomyopathy, Heart failure

## Abstract

Myocarditis, the principal cause of dilated cardiomyopathy and heart failure in young adults, is associated with autoimmunity to human cardiac α-myosin (hCAM) and the DR4 allele of human major histocompatibility II (MHCII). We developed an hCAM-induced myocarditis model in human HLA-DR4 transgenic mice that lack all mouse MHCII genes, demonstrating that immunization for 3 weeks significantly increased splenic T-cell proliferative responses and titres of IgG1 and IgG2c antibodies, abolished weight gain, provoked cardiac inflammation and significantly impaired cardiac output and fractional shortening, by echocardiography, compared to adjuvant-injected mice. Neither cardiac dilatation nor fibrosis occurred at this time point but prolonging the experiment was associated with mortality. Treatment with mixtures of hCAM derived peptides predicted to have high affinity for DR4 significantly preserved ejection fraction and fractional shortening. Our new humanized mouse model of autoimmune cardiomyopathy should be useful to refine hCAM-derived peptide treatment.

## Highlights

• We developed a novel fully-humanized mouse model of autoimmune myocarditis.
• Human leukocyte antigen DR4 transgenic mice were immunized with human cardiac myosin.
• After 3 weeks, mice developed myocarditis and impaired myocardial function.
• A pilot study established the feasibility of peptide immunotherapy in this model.

## 1 Introduction

Myocarditis is the leading cause of heart failure in people under 40 years of age. Viral (frequently Coxsackievirus B3, Parvovirus B19, adenoviruses or herpes viruses), bacterial (e.g. *Corynebacterium diphtheriae*, *Staphylococcus aureus*, *Borrelia burgdorferi* or *Ehrlichia* species) and some parasitic (e.g. *Trypanosoma cruzi*) infections are implicated in acute myocarditis, which becomes chronic and progresses towards dilated cardiomyopathy (DCM) in one fifth of cases [1]. Many chronic patients have autoimmunity to cardiac muscle derived antigens [2], including (in 41%) cardiac specific human α-myosin (hCAM) [3]. As with other autoimmune diseases, there is familial aggregation, autoantibody presence in unaffected relatives [4] and association with specific MHCII genotypes [5]. A recent meta-analysis of 19 independent studies totalling 1378 cases and 10,383 controls demonstrated a statistically elevated frequency of the human leukocyte antigen HLA-DR4 allele [5]. Myocarditis and DCM can be induced in genetically susceptible rodent strains by immunization with hCAM in complete Freund's adjuvant (CFA) but humanized mouse models are needed to evaluate immunotherapies because they bear human rather than mouse MHCII genes. Mice transgenic for the HLA-DQ8 allele on a non-obese, diabetic (NOD) background (so-called NOD.DQ8 mice) develop spontaneous autoimmune myocarditis [6]. However, there is no evidence of increased antibody levels or propensity for DCM in diabetic humans or those carrying the DQ8 allele [5]. Consequently, we studied humanized HLA-DR4 transgenic mice that lack all mouse MHCII genes on a metabolically normal background (henceforth referred to as DR4 mice) [7]. DR4 mice immunized with hCAM developed myocarditis that could be ameliorated by treatment with hCAM-derived peptides.

## 2 Material and methods

### 2.1 Human cardiac α-myosin (hCAM)

Heart tissue was obtained from organ donors giving informed consent under research ethical committee approval (NHS South West – Frenchay Ref No 08/H0107/48) and was stored according to the regulations of the UK Human Tissue Act 2004. hCAM was extracted from homogenised hearts by high-salt precipitation, as described previously [8] and its purity was confirmed by SDS polyacrylamide gel electrophoresis.

#### 2.1.1 Induction of experimental autoimmune myocarditis

DR4 mice (aged 6–8 weeks) were bred by Apitope International NV under licence from the originator, Dr. L Fugger (MRC Human Immunology Unit, University of Oxford, UK). Mouse transgenic for multiple copies of the HLA-DRA*0101 and HLA-DRB1*0401 alleles were generated as described [7] and bred onto the MHCII^Δ/Δ^ strain (which lacks all the mouse MHCII genes [9]) on a C57black/6 background for at least 10 generations. Breeding and experimental procedures were conducted according to the Guide for the care and use of laboratory animals, eighth edition (2011) (http://grants.nih.gov/grants/olaw/guide-for-the-care-and-use-of-laboratory-animals.pdf) and under the approval of UK Home Office licence 30/3195. Purified hCAM (2 mg/ml) was emulsified 1:1 in complete Freund's adjuvant (CFA; Difco #263810) boosted with 8 mg/ml of heat-killed *Mycobacterium tuberculosis H37 Ra* (hkMTB; Difco #231141) and 100 μg myosin/mouse was injected subcutaneously. On day 0 and 2, 200 ng of pertussis toxin (Sigma Aldrich) in 0.5 ml of phosphate buffered saline (PBS) was injected intraperitoneally to each mouse as a second adjuvant [10]. A control group received PBS in the same boosted CFA as well as pertussis toxin.

### 2.2 Echocardiographic analysis of left ventricle function

Anaesthesia was essential to locate the probe accurately and avoid motion during measurements. 3 weeks after immunization, mice were anaesthetised by intraperitoneal injection of 250 mg/kg of tribromoethanol (Avertin; Sigma Aldrich). A left ventricle short-axis view at the papillary muscle level was obtained in M-Mode using a Vevo 770 echocardiography system (Visual Sonics; Toronto, Canada) just before terminating the mice. Because anaesthesia reduces heart rate, all the functional measurements were obtained at between 400 and 500 beats per minute to avoid any artefactual differences.

### 2.3 Histopathological analysis of cardiac inflammation and fibrosis

Hearts were fixed for 24 h in 10% (v/v) formalin in PBS. After embedding in paraffin wax, sections (5 μm) were stained with haemotoxylin and eosin. Images were captured by Aperio slide scanner (Leica Biosystems; Nussloch, Germany) and areas of inflammatory cell infiltration were defined blindly and quantified using Image J (Open Source) software. Masson's trichrome staining was used to detect fibrosis (blue colour).

### 2.4 Anti-hCAM antibody and T-cell proliferation measurements

Serum concentrations of serum CSF2, IFNγ, IL1β, IL2, IL6, IL10 and TNFα were measured by multiplex cytokine analysis (Merck Millipore, Watford, UK) according to the manufacturer's instructions. Serum anti-hCAM antibody levels were measured by ELISA on Nunc Immuno MaxiSorp 96-well flat bottom plates coated with 100 μl of 10 μg/ml purified hCAM in 0.05 M carbonate-bicarbonate buffer (pH 9.0). Bound antibodies were detected with 1:1000 of goat anti-mouse IgG1 or IgG2c secondary antibodies conjugated to alkaline phosphatase (Abcam; Cambridge, UK). For the hCAM-reactive T-cell proliferation assay [11], Spleens were mechanically disrupted in X-VIVO 15 medium (Lonza; Manchester, UK) supplemented with 2 mM l-Glutamine, 100 IU/ml penicillin and 100 μg/ml streptomycin, filtered through a 40 μm cell strainer and the cells collected by centrifugation at 300 × g for 5 min at 4 °C. After treatment with red blood cell lysis buffer (Sigma Aldrich; Poole, UK) cells were washed in PBS and resuspended in supplemented X-VIVO 15 medium. Splenocytes were cultured in 96-well plates (5 × 10^5^ cells/well) for 72 h at 37 °C at 5% CO_2_ in the presence of purified hCAM or concanavalin A. Cells were then pulsed with 0.5 μCi/well of ^3^H-thymidine for 18 h before harvesting onto glass fibre filters (Cox Scientific; Northants, UK) and placing into a 1450 Microbeta Liquid Scintillation Counter (Perkin Elmer; Massachusetts, USA).

### 2.5 In silico prediction and application of hCAM-specific peptides

Linear peptide epitopes binding to DR4 locus with the highest affinity were predicted by the online ProPred, NetMHCII and IEDB algorithms and synthetic peptides (minimum 95% purity) were obtained from GenScript (New Jersey, USA). Their antigenicity was screened on hCAM sensitized splenic T-cells using the forementioned ^3^H-thymidine incorporation assay. The best inducers (pool(1): YHIFYQILSNKKPEL, PHIFSISDNAYQYML, VNPYKWLPVYNAEVV and pool(2): RVQLLHSQNTSLINQ, EATLQHEATAAALRK, KSSLKLMATLFSSYA) were subcutaneously injected in PBS at equal mass ratios (with PBS alone as control) into 6–8 weeks old mice every 2 days starting from 0.1 μg/mouse (total). The dose was escalated 10-fold until 100 μg/mouse was injected 3 times and then every 4 days until the end of the experiment [12].

### 2.6 Statistical analysis

Discrete variables were examined using Fisher's exact test. Kolmogorov-Smirnov tests (n = 5–7) or D'Agostino tests (n > 8) were applied to test normality and data expressed as the mean ± SD (Standard Deviation). A two-tailed unpaired Student's *t*-test or a Wilcoxon non-parametric test was used, as appropriate. For more than two groups, a one-way or two-way ANOVA was performed, followed by a Dunnett or Bonferroni post-test. In all cases, the values were considered significant if the two-tailed probability p < 0.05.

## 3 Results

### 3.1 Effect of hCAM immunization on DR4 mice

**Fig. 1:**
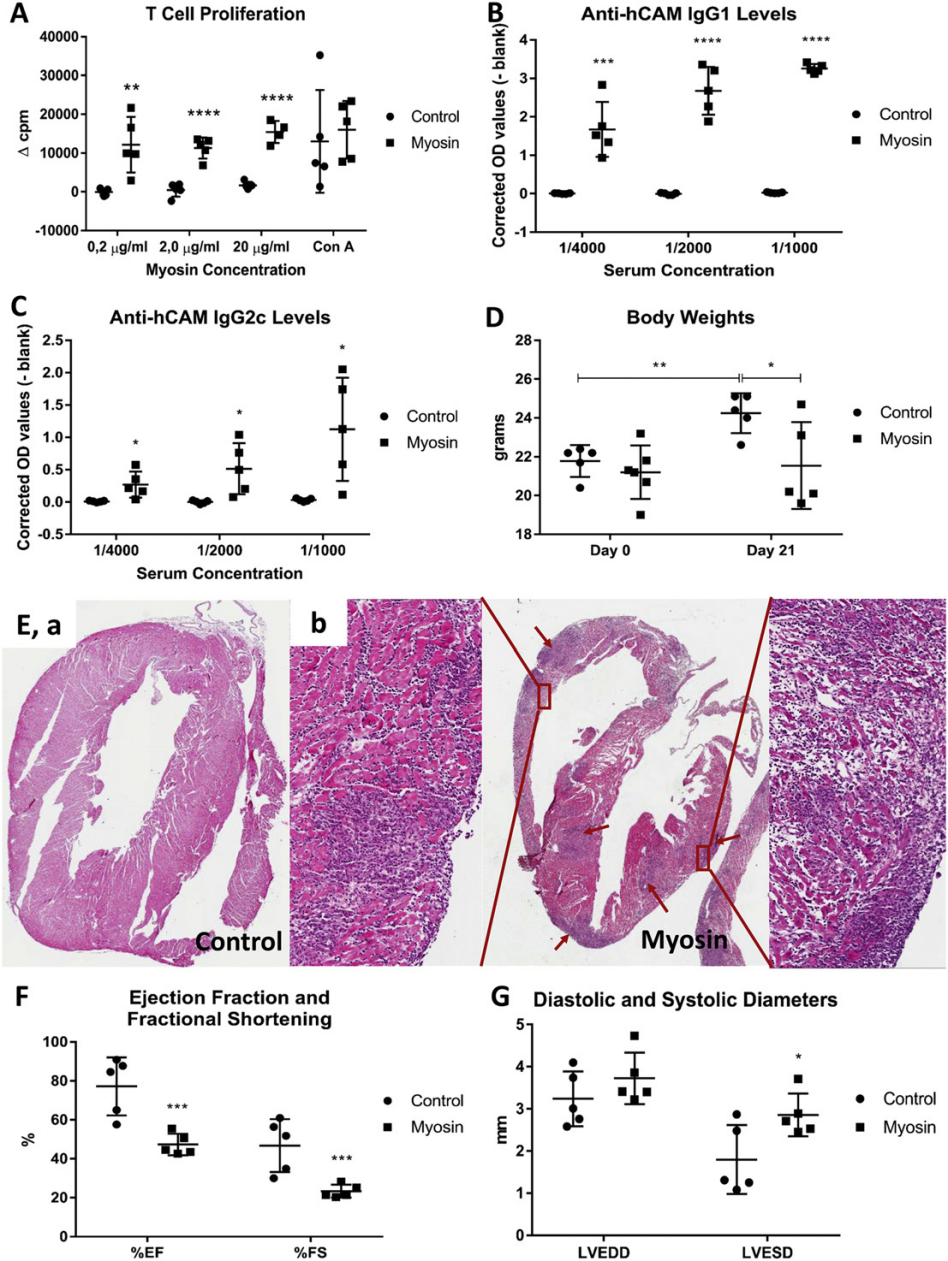
Effects of hCAM immunization on immunological measurements, body weight and myocarditis. (A) Change in thymidine incorporation compared to medium alone controls. Adding hCAM increased spleen cell proliferation after hCAM immunization significantly more than after control immunization (p = 0.022 for 0.2 μg/ml, p = 0.000061 for 2.0 μg/ml and p = 0.0081 for 20 μg/ml hCAM concentration; n = 5). Responses to hCAM were similar to the global T-cell stimulator, ConA (2 μg/ml). (B) Serum IgG1 levels were increased significantly in hCAM compared to control immunized mice (p_max_ = 0.0008; n = 5). (C) Serum IgG2c levels were also significantly increased as compared to control immunized mice (p_max_ = 0.02; n = 5). (D) Mice immunized with hCAM group gained no weight, while vehicle-immunized mice gained 2.4 g and were therefore significantly heavier than hCAM-immunized mice after 3 weeks (p = 0.04; n = 5). (E) Haematoxylin and eosin stained histological sections from hearts of the control immunized mice (panel a) were all clear (0/5), while 5/5 of the hearts (p = 0.0079, Fisher's exact test) from hCAM-immunized mice (panel b) were infiltrated with immune cells (blue areas due to concentrated cell nuclei stained with haematoxylin) and had areas of myocyte necrosis in both left and right ventricles. Echocardiographic measurements were performed 3 weeks after immunization. (F) A significant reduction in percentages of ejection fraction (EF; 44 ± 5 vs 77 ± 3%; p = 0.003; n = 5) and fractional shortening (FS; 22 ± 8 vs 48 ± 2%; p = 0.006; n = 5) were observed in hCAM compared to control immunized mice. (G) A significant increase in left ventricle-end-systolic dimension (LVESD) was also observed (2.8 ± 0.4 vs 1.8 ± 0.2 mm; p = 0.039; n = 5) in hCAM compared to control immunized mice, whereas left ventricle end-diastolic dimension (LVEDD) was not affected.

Addition of hCAM significantly increased proliferation relative to medium controls of splenic T-cells from DR4 mice injected with hCAM/CFA (but not PBS/CFA) and pertussis toxin similar to positive control ConA (Fig. 1A). Evidently, subcutaneous hCAM evoked a strong T-cell mediated immune response. Immunization with hCAM greatly increased serum anti-hCAM IgG1 and IgG2c antibody levels, which were undetectable in the PBS/CFA treated mice (Fig. 1B, C), indicating a strong B-cell response also. When serum concentrations of CSF2, IFNγ, IL1β, IL2, IL6, IL10 and TNFα were measured by multiplex cytokine analysis, only IL6 concentration was significantly increased from 58 ± 8 to 133 ± 28 pg/ml (p = 0.025, n = 5). DR4 mice gained 2.4 g in weight 3 weeks after PBS/CFA immunization but hCAM-immunized mice stopped gaining weight and became significantly lighter than PBS/CFA controls (Fig. 1D), suggestive of generalised malaise. No PBS/CFA treated mice (0/5) had histological evidence of leukocyte infiltration or myocyte damage (Fig. 1E, a), indicating absence of spontaneous autoimmune myocarditis at 9–11 weeks. By contrast, all hCAM-immunized mice (5/5) showed histological evidence of inflammation and myocardial necrosis (p = 0.0079, Fisher's exact test). This affected both atria and ventricles (e.g. Fig. 1E, b) and averaged approximately 30% of the area of sections. None of the hearts from PBS/CFA injected control mice or myosin injected animals showed any fibrosis using Masson's trichrome staining (0/5). In a separate study, 3/5 immunized and 0/5 control mice died when the experiment lasted longer than 3 weeks. More mortality data were not collected for ethical reasons. In another pilot study, myocarditis did not occur in the absence of pertussis toxin. Most importantly, hCAM immunization plus pertussis impaired left ventricular function, significantly decreasing ejection fraction (44 ± 5 vs 77 ± 3%; p = 0.003; n = 5) and fractional shortening (22 ± 8 vs 48 ± 2%; p = 0.006; n = 5) (Fig. 1F) and increasing end systolic dimensions (2.8 ± 0.4 vs 1.8 ± 0.2 mm; p = 0.039; n = 5, Fig. 1G) compared to PBS/CFA treated mice. However, end-diastolic dimensions were not significantly increased, despite a weak trend (p = 0.25, n = 5), implying no cardiac dilatation (Fig. 1G). Hence hCAM immunization caused autoimmune myocarditis with impairment of left ventricular function, which did not progress to significant DCM or fibrosis after 3 weeks.

### 3.2 Feasibility study of hCAM-specific peptide treatment

**Fig. 2:**
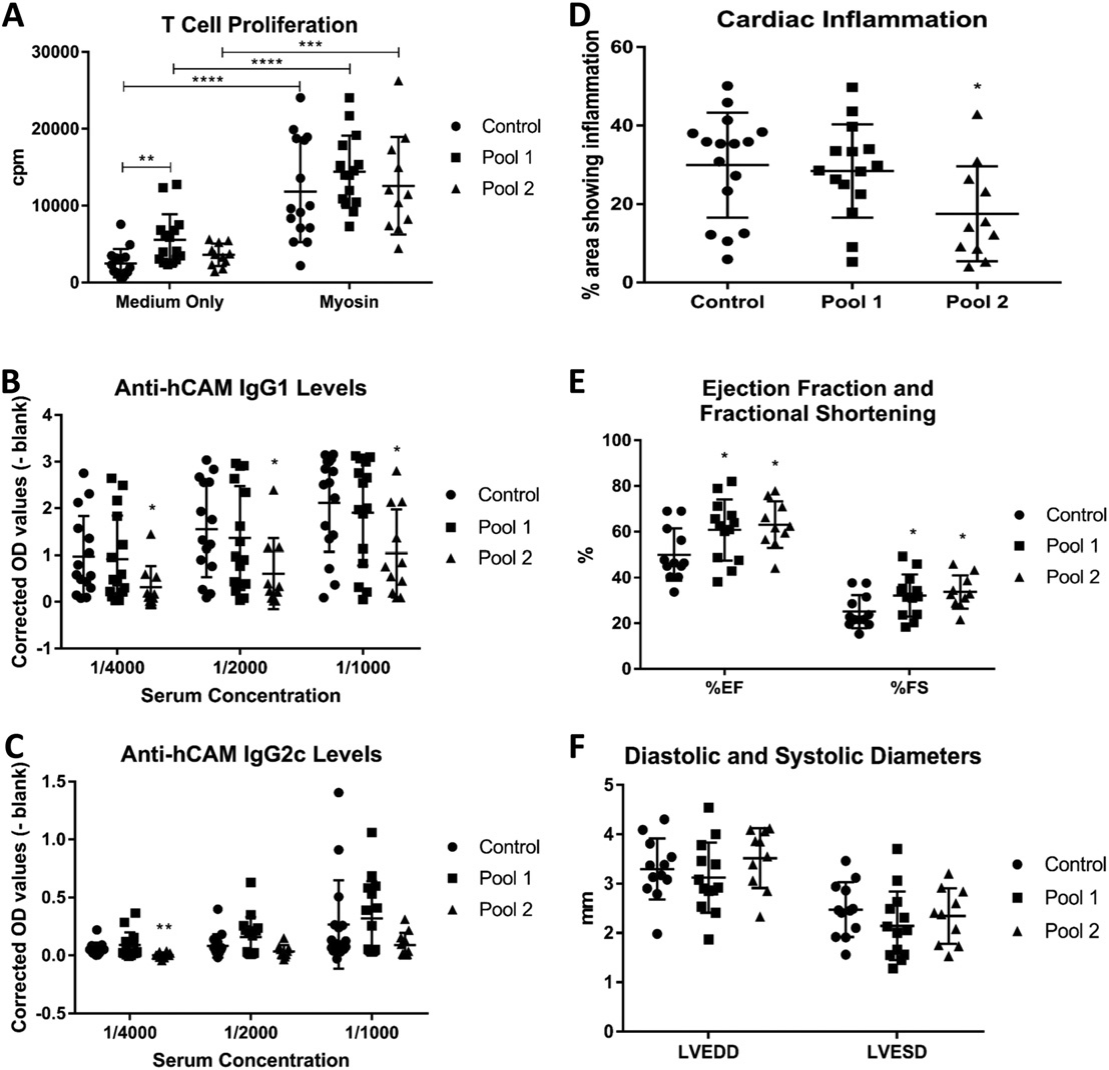
Effect of hCAM-derived peptide pre-treatment. (A) Basal spleen cell proliferation was increased by pool(1) peptide (n = 15) compared to vehicle treatment (n = 15, p = 0.0045) but not with pool(2) (n = 11). Addition of hCAM (20 μg) stimulated splenocyte proliferation to a similar extent after treatment with vehicle, pool(1) or pool(2) peptides. (B) Serum anti-hCAM IgG1 levels were decreased significantly after treatment with pool(2) peptides (n = 11), compared to the vehicle treated group (n = 15), at all dilutions (p_max_ = 0.036), while no effect was recorded with pool(1) peptides (n = 15). (C) Serum anti-hCAM IgG2c levels were also decreased significantly with only pool(2) peptides at 1/4000 dilution (p = 0.0014; n = 11 vs 15; Wilcoxon test was applied due to non-Gaussian distribution). (D) The area of sections occupied by leukocyte infiltration and myocyte damage was reduced significantly by pool(2) treatment (17.5 vs 29.9%; p = 0.021; n = 11), whereas pool(1) treatment (n = 15) had no significant effect compared to vehicle treated controls (n = 15). (E) Both peptide treatments significantly increased EF (p = 0.011, n = 10 for pool(2); p = 0.041, n = 13 for pool(1)) and FS (p = 0.011, n = 10 for pool(2); p = 0.044, n = 13 for pool(1)) relative to vehicle injected controls (n = 12) 3 weeks after hCAM immunization. (F) LVEDD and LVESD were not significantly affected by peptide treatments.

Autoantigen-derived peptides with high affinities for MHCII can provoke immunomodulation and hence desensitize T-cell responses in models of other autoimmune diseases [13]. To establish the feasibility of this approach to reduce myocarditis in DR4 mice, ten candidate 15 amino acid long peptides with predicted high affinity for the DR4 allele were identified using online prediction tools. The six that stimulated T-cell proliferation in splenocytes from hCAM-immunized DR4 mice were chosen for further study. Subcutaneous pre-treatment and concurrent treatment with two pools of three peptides each (specified in Materials and methods) followed an escalating dose schedule established previously for experimental autoimmune encephalomyelitis [12]. Base-line proliferation of splenic T-cells from hCAM-immunized mice was increased and the response to hCAM was not significantly affected after treatment with pool(1) peptides, implying, unexpectedly, that pool(1) stimulated rather than desensitised T-cell responses (Fig. 2A). Pool(1) peptides did not raise serum anti-hCAM IgG1 or IgG2c antibodies in control mice, nor did they reduce levels in hCAM-immunized mice (Fig. 2B, C). Pool(2) peptides did not significantly affect basal or hCAM-stimulated splenic T-cell proliferation (Fig. 2A) but they significantly decreased serum IgG1 and IgG2c antibody levels in hCAM-immunized mice (Fig. 2B, C). Hence pool(2) produced a clear immunomodulatory effect that appeared insufficiently pronounced to significantly affect T-cell responses measured by our method. Serum concentrations of CSF2, IFNγ, IL1β, IL2, IL6, IL10 and TNFα were unaffected by pool(1) or pool(2) peptide treatments (n = 9/10 versus n = 10 myosin injected controls). PBS-injected hCAM-immunized animals did not gain weight (25 ± 3 to 26 ± 3 g), similar to the situation with immunization alone. Weight gain was not restored by pool(1) or pool(2) peptide treatments (24 ± 3 to 24 ± 3 and 25 ± 3 to 27 ± 3 g, respectively), perhaps owing to the added trauma of repeated subcutaneous injections. PBS-treated hCAM-immunized mice had 30 ± 13% of their myocardium showing inflammatory changes by histopathology. Myocarditis was not reduced by pool(1) peptides (28 ± 12%) but pool(2) treatment caused a significant reduction (18 ± 12%; p = 0.021; n = 11) (Fig. 2D). Fibrosis was detected (< 5% of the total area) in 2/10 myosin injected control mice 2/10 pool(1) and 1/10 pool(2) treated mice. PBS-injected, hCAM-immunized mice showed similar cardiac function to untreated hCAM-immunized mice (Fig. 2E, F compared to Fig. 1F, G). Treatment with either pool(1) or pool(2) peptides significantly improved ejection fraction (p = 0.041, n = 13 for pool(1); p = 0.011, n = 10 for pool(2)) and fractional shortening (p = 0.044, n = 13 for pool(1); p = 0.011, n = 10 for pool(2)) of hearts from hCAM-immunized mice (Fig. 2E). Values were returned towards those seen in unimmunized mice (Fig. 1F). Both pools also decreased end systolic dimension but these effects were not significant (Fig. 2F). Neither pool decreased end-diastolic dimensions (Fig. 2F).

## 4 Discussion

We describe a new humanized model of autoimmune myocarditis, measured histologically, and impaired cardiac function, measured by ultrasonography, based on hCAM recognition by the HLA-DR4 allele, which is overrepresented in chronic myocarditis patients. Serum cytokines proved to be an insensitive marker of disease and future studies should rather investigate myocardial cytokine levels. Myocarditis did not progress to significant DCM or fibrosis after 3 weeks. However, longer exposure led to increased mortality, which precluded later measurements. Pertussis toxin treatment was added for its selective mitogenic effect on Thelper1 lymphocytes, as established in other autoimmunity models [10]. A recent publication suggested that hCAM causes autoimmunity in NOD.DQ8 mice because it is not expressed in the thymus, the critical organ responsible for central tolerance among developing T-cells [14]. Humans also do not express hCAM in the thymus, suggesting a paradigm for the pathogenesis of myocarditis in both species [14]. Interestingly, DR4 mice on a normal metabolically background did not develop myocarditis at the same age as DQ8 mice on a NOD background [6]. The contribution of HLA genotype and metabolic background to this discrepancy are worthy of future study but beyond the present scope.

Immuno-dominant peptides might be considered for immunomodulation therapy, particularly in autoimmune myocarditis because there is an opportunity to treat DR4 positive acute myocarditis patients before the development of chronic myocarditis or DCM. Models for other autoimmune diseases show that immunotherapy is more potent in preventing than treating autoimmunity [15]. To identify the most effective peptides, it would be necessary (albeit far beyond our present resources) to screen the whole hCAM sequence for dominant epitopes as they evolve over time by using overlapping peptides. To demonstrate the feasibility of using our mice for this approach we used peptide pools with predicted high affinity for DR4. Disappointingly, neither of the pools we studied reduced the T-cell proliferation response to hCAM. Peptide therapy can induce tolerance owing to antigen-specific T-cell deletion or anergy. However, stimulated in situ differentiation of extra-thymic, inducible, IL-10 producing regulatory T cells, which may be Foxp3 negative (Tr1 cells) or positive, is the most established mechanism [13]. All of these changes should result in reduced antigen-specific T-cell proliferation and reduced T-cell help to B-cells leading to reduced antibody production. In our feasibility study, the peptides were not fully optimised and hence suppression of T-cell proliferation was not observed. B-cell help probably involves the highest affinity T-cell clones and therefore may be a more sensitive indicator of immunomodulation. In addition, T-cells should ideally be derived from the affected organ or the nearby draining lymph nodes, which may be advantageous for future studies. Given these limitations, the mechanisms were not pursued further. Optimisation of peptides is clearly necessary also before any clinical translation could be envisioned. More positively, we demonstrated, for the first time in any autoimmune myocarditis model, that at least for one pool of peptides ameliorated histological measures of inflammation (Fig. 2D) and that both peptide pools improved cardiac function (Fig. 2E). These pilot studies therefore demonstrate the utility of our new model for refining immunomodulatory peptide therapy.

## Disclosures

DCW receives a consultancy fee from Apitope International NV, which supported this research, in part, through a grant in aid.
